# Accuracy of Ultrasonography vs. Elastography in Patients With Non-alcoholic Fatty Liver Disease: A Systematic Review

**DOI:** 10.7759/cureus.29967

**Published:** 2022-10-06

**Authors:** Prabhitha Geethakumari, Prathima Kampa, Rakesh Parchuri, Renu Bhandari, Ali R Alnasser, Aqsa Akram, Saikat Kar, Fatema Osman, Ghadi D Mashat, Hadrian Hoang-Vu Tran, Neway A Urgessa, Ann Kashmer Yu

**Affiliations:** 1 Internal Medicine, California Institute of Behavioral Neurosciences & Psychology, Fairfield, USA; 2 Internal Medicine/Family Medicine, California Institute of Behavioral Neurosciences & Psychology, Fairfield, USA; 3 General Surgery, California Institute of Behavioral Neurosciences & Psychology, Fairfield, USA; 4 Pediatrics, California Institute of Behavioral Neurosciences & Psychology, Fairfield, USA; 5 Research, California Institute of Behavioral Neurosciences & Psychology, Fairfield, USA

**Keywords:** diagnostic test accuracy, non-alcoholic steatohepatitis (nash), diagnostic elastography, ultrasonography (usg), nonalcoholic fatty liver disease (nafld)

## Abstract

Ultrasonography and elastography are the most widely used imaging modalities for diagnosing non-alcoholic fatty liver disease. This study aimed to assess and compare the diagnostic accuracy in patients with non-alcoholic fatty liver disease/non-alcoholic steatohepatitis. This systematic review was based on the Preferred Reporting Items for Systematic Reviews and Meta-Analyses (PRISMA) guidelines. A systematic search was done for the past seven years using Pubmed, Pubmed Central, Cochrane, and Google Scholar databases on Jun 29, 2022. Studies were included based on the following predefined criteria: observational studies, randomized controlled trial (RCT), comparative studies, studies using liver biopsy or MRI proton density fat fraction (MRI PDFF) as a reference standard, ultrasonography, and elastography with measures of their diagnostic accuracy like sensitivity (SN), specificity (SP), area under the receiver operating characteristic (AUROC) curve, and English language. The data were extracted on a predefined template. The final twelve eligible studies were assessed using the quality assessment of diagnostic accuracy tool (QUADS-2). Most studies focused on elastography techniques, and the remaining focused on quantitative ultrasonography methods like the controlled attenuation parameter (CAP) and attenuation coefficient (AC). Only one study was available for the evaluation of qualitative ultrasonography. MRI was generally found superior to other diagnostic tests for determining liver stiffness through magnetic resonance elastography (MRE) and steatosis through MRI PDFF. Data assessing the comparative diagnostic accuracy of the two tests were inconclusive.

## Introduction and background

The prevalence of non-communicable diseases (NCDs) has sharply risen in recent decades, and metabolic syndrome has primarily been at the forefront. The high-calorie, low-fiber food consumption, sedentary lifestyle, and increasing use of automated machines in our day-to-day lives have significantly contributed to it [1]. The complications of NCD are widely varied, and one which mainly targets the liver is non-alcoholic fatty liver disease (NAFLD).

NAFLD comprises a spectrum of liver diseases specifically seen in non-alcoholic patients, characterized by histological changes ranging from simple hepatic steatosis (fatty liver) to more progressive steatosis, ballooning, and inflammation (non-alcoholic steatohepatitis (NASH)) [2]. The global prevalence of NAFLD is 25% [3]. Only 20-30% of cases with NAFLD progress to NASH [4].

NASH/NAFLD is associated with an increased risk of developing cirrhosis, cardiovascular diseases, and cancer [3,5]. Most patients with NAFLD remain asymptomatic until irreversible damage already occurs in the liver. Hence diagnosing the disease is paramount in delaying progression and preventing complications. The definitive diagnostic method for NASH remains liver biopsy, but its limitations include invasiveness, sampling, and complications [6]. So research focused on non-invasive diagnostic modalities has led to the emergence of newer imaging techniques, including elastography, controlled attenuation parameters, serum biomarkers like cytokeratin, aminotransferases, and scoring systems [7]. However, ultrasonography remains the most widely used diagnostic modality owing to its low cost and availability worldwide, particularly in developing nations [8]. The poor inter-observer agreement and its highly subjective nature are some of the limitations of conventional ultrasound [9]. The emergence of newer techniques like elastography for measuring liver stiffness has provided a much-needed alternative non-invasive method for assessing liver fibrosis [10]. None of the studies in the current literature compare the diagnostic accuracy of these imaging techniques for a complex disease like NAFLD/NASH. This systematic review aims to address this gap and assess the accuracy of ultrasonography and elastography in diagnosing patients with NAFLD/NASH.

## Review

Methods

This systematic review was conducted according to The Preferred Reporting Items for Systematic Reviews and Meta-Analyses (PRISMA) 2020 guidelines [11].

Eligibility criteria

The studies which fulfilled the following criteria were included: 1) randomized controlled trials, clinical trials, observational studies, meta-analysis, traditional reviews, systematic reviews, comparative studies, and technical reports published between 2013-2022; 2) articles in the English language; 3) free full-text articles; 4) studies that included adults (age > 18 years); 5) diagnosed with NASH or NAFLD either histologically or clinically; 6) ultrasonography or elastography for diagnosis of NASH; 7) data regarding sensitivity, specificity, or area under the receiver operating characteristic (AUROC) curve was available, and 8) reference standard test was either liver biopsy or magnetic resonance proton density fat fraction (MRI PDFF). The studies which fulfilled the following criteria were excluded: 1) editorial, observational study veterinary, retracted publication; 2) articles before 2013, and also those with only abstract; 3) articles in a language other than English; 4) patients < 18 years old; 5) patients diagnosed with cirrhosis due to causes other than NAFLD; and 6) studies using any reference standard other than the above mentioned.

Search strategy

A systematic search was conducted by scouring the following databases: PubMed, Google Scholar, Cochrane, and PubMed Central. The last search date for all databases was on June 29, 2022. The keywords and the heading terms used were based on the previous literature and through Medical Subject Headings (Mesh), depending on the database used, as seen in Table 1.

**Table 1:** The search strategy used in this systematic review

Database	Keyword	Search strategy	Filter	Results
Pubmed	NASH: Non-alcoholic steatohepatitis, Non-alcoholic fatty liver disease, NAFLD, Non alcoholic steatohepatitides, Non alcoholic fatty liver, NASH Elastography: Elastography, Elasticity imaging technique, Elastographies, vibro-acoustography, Tissue Elasticity Imaging Ultrasound: Ultrasound, Ultrasonography, Ultrasound imaging, imaging non-invasive, Ultrasonographic imaging, Diagnostic ultrasound	#1 Search: NASH:Non alcoholic steatohepatitis OR Non alcoholic fatty liver disease OR NAFLD, Non alcoholic steatohepatitides OR Non alcoholic fatty liver OR NASH OR( "Non-alcoholic Fatty Liver Disease/diagnosis"[Mesh] OR "Non-alcoholic Fatty Liver Disease/diagnostic imaging"[Mesh] OR "Non-alcoholic Fatty Liver Disease/pathology" [Mesh]		42189
		#2 Search: Elastography OR Elasticity imaging technique OR Elastographies, vibro-acoustography OR Tissue Elasticity Imaging OR ( "Elasticity Imaging Techniques/standards"[Mesh] OR "Elasticity Imaging Techniques/statistics and numerical data"[Mesh] OR "Elasticity Imaging Techniques/therapeutic use"[Mesh] )		16575
		#3 Search: Ultrasound: Ultrasound OR Ultrasonography OR Ultrasound imaging OR imaging non invasive OR Ultrasonographic imaging OR Diagnostic ultrasound OR ( "Ultrasonography/standards"[Mesh] OR "Ultrasonography/statistics and numerical data"[Mesh] OR "Ultrasonography/therapeutic use"[Mesh] )		1881382.
		#4 Search #1 AND #2 AND #3		1068
		#5 Search#1 AND #2 AND #3	Free full text, Comparative Study, Meta-Analysis, Observational Study, Randomized Controlled Trial, Review, Systematic Review, Technical Report, English, from 2013 - 2022	152
Google scholar	NASH, USG, Elastography	NASH AND USG AND Elastography	2017- 2022	2660
		NASH AND USG AND elastography Free full text, Meta-Analysis, Observational Study, Randomized Controlled Trial, review, systematic review in the last 5 years		404
Cochrane library		#1 Non-alcoholic steatohepatitis OR Non-alcoholic fatty liver disease OR NAFLD, Non-alcoholic steatohepatitides OR Non-alcoholic fatty liver OR NASH,		4752
		#2 Elastography OR Elasticity imaging technique OR Elastographies, vibro-acoustography OR Tissue Elasticity Imaging, Results		878
		#3 Ultrasound OR Ultrasonography OR Ultrasound imaging OR imaging non invasive OR Ultrasonographic imaging OR Diagnostic ultrasound		48223
		#4= #1 AND #2 AND #3	Cochrane reviews, Cochrane protocols, trials, and date of publishing between January 2017 and July 2022	60
Pubmed Central		#1 Search (Non alcoholic steatohepatitis OR Non alcoholic fatty liver disease OR NAFLD, Non alcoholic steatohepatitides OR Non alcoholic fatty liver OR NASH OR( "Non-alcoholic Fatty Liver Disease/diagnosis"[Mesh] OR "Non-alcoholic Fatty Liver Disease/diagnostic imaging"[Mesh] OR "Non-alcoholic Fatty Liver Disease/pathology"[Mesh] ))		90210
		#2 Search (Elastography OR Elasticity imaging technique OR Elastographies, vibro-acoustography OR Tissue Elasticity Imaging OR ( "Elasticity Imaging Techniques/standards"[Mesh] OR "Elasticity Imaging Techniques/statistics and numerical data"[Mesh] OR "Elasticity Imaging Techniques/therapeutic use"[Mesh] ))		48054
		#3 Search (: Ultrasound OR Ultrasonography OR Ultrasound imaging OR imaging non invasive OR Ultrasonographic imaging OR Diagnostic ultrasound OR ( "Ultrasonography/standards"[Mesh] OR "Ultrasonography/statistics and numerical data"[Mesh] OR "Ultrasonography/therapeutic use"[Mesh] ))		1099339
		#4 diagnostic accuracy		718174
		#7 Search (sensitivity and specificity)		804261
		#8 Search ((((#4) AND #5) AND #6) AND #7) AND #8		1831
		#9 Search ((((#4) AND #5) AND #6) AND #7) AND #8	published in the last five years	1198
		#10 Search ((((#4) AND #5) AND #6) AND #7) AND #8	Open access; published in the last five years	963

All the references collected from the search strategy were arranged alphabetically using Microsoft Excel 2019. The duplicates were first removed, and the remaining articles were further reviewed through titles and abstracts to exclude the irrelevant ones. It was followed by screening full-text articles to narrow down the included studies according to the eligibility criteria.

Risk of bias

The final articles which remained after the screening process were assessed for the risk of bias using a quality assessment tool: Quality Assessment of Diagnostic Accuracy Studies (QUADAS 2) [12]. The signalling questions and the risk of bias in each domain were assessed and the responses were marked as yes, no, or unclear. The details of the QUADAS 2 tool are given in Table 2.

**Table 2:** QUADAS 2 tool showing the various domains and signaling questions

Domain 1 - patient selection
A: Risk of bias
1) Was a consecutive or random sample of patients enrolled?
2) Was a case-control design avoided?
3) Did the study avoid inappropriate exclusions?
Could the selection of patients have introduced bias?
B: Concerns regarding applicability
1) Is there concern that the included patients do not match the review question?
Domain 2 - Index test
A: Risk of bias
1) Were the index test results interpreted without knowledge of the results of the reference standard?
2) If a threshold was used, was it pre-specified?
Could the conduct or interpretation of the index test have introduced bias?
B: Concerns regarding applicability
1) Is there concern that the index test, its conduct, or interpretation differ from the review question?
Domain 3- Reference standard
A: Risk of bias
1) Is the reference standard likely to correctly classify the target condition?
2) Were the reference standard results interpreted without knowledge of the results of the index test?
Could the reference standard, its conduct, or its interpretation have introduced bias?
B: Concerns regarding applicability
1) Is there concern that the target condition as defined by the reference standard does not match the review question?
Domain 4- Flow and timing
1) Was there an appropriate interval between index test(s) and reference standard?
2) Did all patients receive a reference standard?
3) Did patients receive the same reference standard?
4) Were all patients included in the analysis?
Could the patient flow have introduced bias?

Data extraction and assessment

The duplicates were first removed from the studies collected. The studies were further filtered out by a screening process of titles and abstracts by two reviewers independently. The same reviewers also did the quality assessment of the studies, and in cases of discrepancies, a third reviewer helped to reach a consensus. Information regarding the author, study design, population characteristics, index, and reference tests were extracted from the studies and formulated in a table. The parameters of diagnostic accuracy, including sensitivity (SN), specificity (SP), positive predictive value (PPV) and negative predictive value (NPV), and AUROC curve of the included studies, were recorded and tabulated. Information regarding the cut-off points for the relevant tests and the sample population at various stages of steatosis and fibrosis were also included. Meta-analysis was not done due to the clinical heterogeneity in the included studies and the few studies identified for individual tests. Hence this systematic review presents the outcome, applications, and limitations of the included studies in the form of a narrative synthesis. 

Results

Study Selection and Quality Assessment

**Figure 1:**
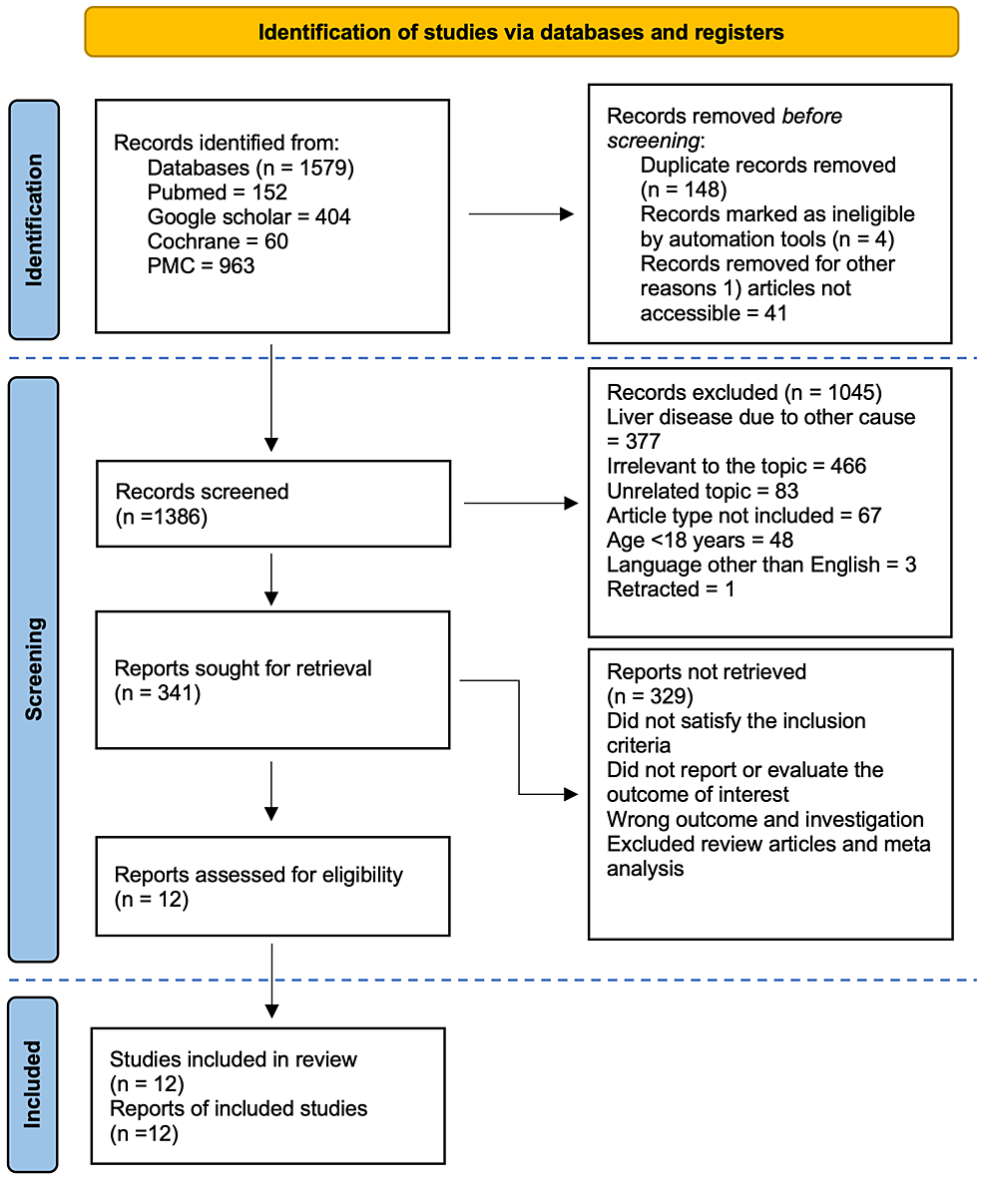
Flow chart showing the study selection and screening process PMC- PubMed Central

The search of the databases yielded a total of 1,579 potentially relevant articles. After the removal of inaccessible articles and duplicates, 1,386 articles remained. These articles were first screened by titles and abstracts to filter out the irrelevant ones by following the eligibility criteria, which led to the exclusion of 1,045 articles. The remaining articles were screened by full text to include only those that fully satisfied the inclusion criteria, leading to twelve studies. These were assessed for quality analysis. The study selection process and screening are given in the form of a flowchart in Figure 1.

The quality assessment of the final studies was done using QUADAS 2 tool, which mainly evaluates the studies on four key domains: patient selection, index test, reference standard, and flow and timing. The risk of bias and applicability were assessed in these domains, and the results are presented in Table 3.

**Table 3:** Risk of bias and concerns regarding the applicability of the studies included in the review

Study	RISK OF BIAS				APPLICABILITY CONCERNS		
	Patient selection	Index test	Reference standard	Flow and timing	Patient selection	Index test	Reference standard
Park et al., 2017 [13]	unclear	unclear	low	low	low	low	low
De Lucia Rolfe et al., 2018 [14]	high	low	low	unclear	unclear	low	low
Taibbi et al., 2020 [15]	low	unclear	low	unclear	low	low	low
Ferraioli et al., 2020 [16]	low	low	low	low	low	low	low
Beyer et al., 2021 [17]	unclear	low	low	unclear	low	low	low
Ogino et al., 2021 [18]	unclear	unclear	low	unclear	low	low	low
Qu et al., 2021 [19]	unclear	low	low	low	low	unclear	low
Sharpton et al., 2021 [20]	low	low	low	unclear	low	unclear	low
Tang et al., 2021 [21]	unclear	low	low	low	low	low	low
Zhang et al., 2021 [22]	unclear	unclear	low	unclear	low	low	low
Imajo et al., 2022 [23]	unclear	unclear	low	low	low	low	low
Ali et al., 2022 [24]	unclear	low	low	unclear	unclear	low	low

Study characteristics

Out of the 12 studies in this systematic review, one focused on qualitative or conventional Ultrasonography, and four focused on quantitative ultrasound parameters like the controlled attenuation parameter (CAP) and attenuation coefficient (AC). Seven focused on elastography techniques like transient elastography (TE), point shear wave elastography (pSWE), vibration-controlled transient elastography (VCTE), and magnetic resonance elastography (MRE). The studies included 1,701 participants and all the studies except for two used liver biopsy as the reference standard. The two exceptions used MRI proton density fat fraction (MRI-PDFF) as the reference standard. One study used a bariatric population as the study cohort, and another involved participants from the geriatric age group as the study cohort. The study characteristics, including the population characteristics, index, and reference tests, are given in Table 4.

**Table 4:** Main characteristics of the diagnostic accuracy studies included in the review BMI- Body mass index, MRE- Magnetic resonance elastography, TE- Transient Elastography, CAP- Controlled attenuation parameter, USG- Ultrasonography, NAFLD- Non-alcoholic fatty liver disease, NASH- Non-alcoholic steatohepatitis, pSWE-  Point shear wave elastography, ATI-Pen – Attenuation imaging penetration, ATI-Gen- Attenuation imaging general, MRS- Magnetic resonance spectroscopy, MRI PDFF- Magnetic resonance imaging Proton density fat fraction, UGAP- Ultrasound guided attenuation parameter, LSM- liver stiffness measurement, UAP- Ultrasound attenuation parameter, VCTE- Vibration controlled transient elastography, SWE- Shear wave elastography

Author, year	Study design and setting	Sample size	%female/ %male	BMI	Age	Index test	Reference standard	Target condition	Fund- ing
Park et al., 2017 [13]	Cross-sectional study, tertiary medical center	104	57/43	30.4	50.8	MRE, TE, CAP	Liver biopsy	NAFLD/ NASH	Yes
De Lucia Rolfe et al., 2018 [14]	Clinical trial	72	42/58	26.6± 3.8	72 ± 2.5	USG	MRS	NAFLD/ NASH	Yes
Taibbi et al., 2020 [15]	Prospective study, tertiary center	46	58.7/41.3	29.4± 4.5	54.7± 9.1	TE, pSWE	Liver biopsy	NAFLD/ NASH	unclear
Ferraioli et al., 2020 [16]	Cross-sectional study	72	57/43	30.8 ± 5.0	52.5 ± 14.9	CAP, ATI-Gen, ATI-Pen	MRI PDFF	NAFLD/ NASH	Yes
Beyers et al., 2021 [17]	Retrospective analysis of two independent studies	580	60/40	31.39 [26.8–36.8]	56	CAP	Liver biopsy	NAFLD/ NASH	Yes
Ogino et al., 2021 [18]	Retrospective study, Omori Medical Center	84	63/37	29.0 ± 4.3	54 ± 13	UGAP	Liver biopsy	NAFLD/ NASH	unclear
Qu et al., 2021 [19]	Multicenter study, clinical trial	237	38/62	25.65 ± 4.27	41.71 ± 12.49	UAP and LSM by Fibrotouch	Liver biopsy	NAFLD/ NASH	Yes
Sharpton et al., 2021 [20]	Prospective cohort study, University of California San Diego	114	54.4/45.6	31.2 (29–34)	55 (45–64)	2D SWE and VCTE	Liver biopsy	NAFLD/ NASH	Yes
Tang et al., 2021 [21]	Retrospective analysis of three prospective studies	91	39.6/60.4	30.9 ± 5.1	50.4 ± 14.3	MRE	Liver biopsy	NAFLD/ NASH	Yes
Zhang et al., 2021 [22]	Prospective single-center cohort	100	46/54	31.6 ± 4.7	51.8 ± 12.9	MRE, SWE	Liver biopsy	NAFLD/ NASH	Yes
Imajo et al., 2022 [23]	Clinical trial, Yokohama university hospital	201	52.7/47.2	27.1 (25.2–30.8)	61.0 (51.0–71.0)	MRE, VCTE, SWE	Liver biopsy	NAFLD/ NASH	Yes
Ali et al., 2022 [24]	Prospective study, bariatric clinic	167	83.2/16.8	48	46	TE	Liver biopsy	NAFLD/ NASH	Yes

The diagnostic accuracy parameters of the qualitative and quantitative ultrasonography tests evaluated in the studies, including sensitivity, specificity, and AUROC, are given in Table 5.

**Table 5:** The diagnostic accuracy measures of the included studies evaluating the qualitative and quantitative parameters of the ultrasound SN- Sensitivity, SP- Specificity, PPV- Positive predictive value, NPV- Negative predictive value, AUC- Area under the curve, USG- Ultrasonography, CAP- Controlled attenuation parameter, ATI- Pen- Attenuation imaging penetration, ATI-Gen- Attenuation imaging general, UAP- Ultrasound attenuation parameter, AC- Attenuation Coefficient, MRI PDFF- Magnetic resonance imaging Proton density Fat Fraction, S- Steatosis, F-Fibrosis.

Study	Severity of fatty liver	Index test	Threshold	SN	SP	PPV	NPV	AUC	p
De Lucia Rolfe et al., [14]	Any degree of steatosis(S)	USG	-	96% ( 87– 99.6%)	94% (73– 100%)	98% ( 90–100%)	-	-	-
Beyers et al., [17]	Steatosis ≥ 1 (n = 225)	CAP	268.5 (dB/m)	0.89	1	1	0.92	0.95 (0.91– 0.99	-
	≥ 2 (n = 139)		308.5(dB/m)	0.78	0.41	0.56	0.65	0.6(0.55– 0.65)	-
	≥ 3 (n = 80)		337.8(dB/m)	0.61	0.59	0.24	0.87	0.63(0.57- 0.70)	-
Ferraioli et al., [16]	S0 vs S1–S3 (MRI‐PDFF >5%)	ATI-Pen	>0.69 dB/cm/MHz	78.6 (63.2–89.7)	95.8 (78.9– 99.9)	97.1 (84.4–99.9)	71.9 (53.3– 86.3)	0.90 (0.81– 0.96)	-
		ATI-Gen	>0.62 dB/cm/MHz	81.1 (64.8–92.0)	95.6 (78.1– 99.9)	96.8 (82.9–99.9)	75.9 (56.1– 89.9)	0.92 (0.82– 0.98)	-
		CAP	>273 dB/m	80.0 (65.4–90.4)	83.3 (62.6– 95.3)	90.0 (76.1–97.4)	69.0 (48.8– 85.0)	0.85 (0.74– 0.92)	-
Ogino et al., [18]	≥S1	AC	0.60 mm	86.7	88.9	98.5	44.4	0.94	-
	≥S2		0.71 mm	85.7	91.8	88.2	90	0.95	-
	≥S3		0.72 mm	85.7	80	46.2	96.6	0.88	-
Qu et al., [19]	S ≥ S1 (≥5%)	UAP	244 dB/m	0.79 (0.73–0.86)	0.86 (0.79– 0.93)	0.89 (0.83–0.94)	0.74 (0.66– 0.82)	0.88 (0.84– 0.92)	-
	S ≥ S2 (≥34%)		269 dB/m	0.87 (0.80–0.94)	0.90 (0.85– 0.94)	0.82 (0.74–0.90)	0.93 (0.88– 0.97)	0.93 (0.89– 0.97)	-
	S = S3 (≥67%)		296 dB/m	0.89 (0.78–1.00)	0.83 (0.78– 0.88)	0.41 (0.29–0.53)	0.98 (0.96– 1.00)	0.88 (0.81– 0.94)	-
Park et al., [13]	S Grade 1-3 (n=71) versus Grade 0 (n=7)	MRI-PDFF	3.71	95.8	100	100	70	0.99 (0.98– 1.00)	PDFF vs CAP = 0.0091
		CAP USG	261	71.8	85.7	98.1	23.1	0.85 (0.75– 0.96)	

The diagnostic accuracy parameters of the different elastography techniques are given in table 6.

**Table 6:** The diagnostic accuracy measures of the studies included in this review evaluating the different elastography techniques SN- Sensitivity, SP- Specificity, PPV- Positive predictive value, NPV- Negative predictive value, AUC- Area under the curve, S- Steatosis, F-Fibrosis, MRE- Magnetic resonance elastography, TE- Transient Elastography, LSM- Liver stiffness measurement, VCTE- Vibration controlled transient Elastography, 2D-SWE- Two dimensional Shear wave elastography, **Study - Zhang et al.- results/values=sensitivity>90% (specificity >90%)*, SWE- Shear wave elastography, SWE 10- Shear wave elastography with 10 measurements, SWE 5- Shear wave elastography with first five measurements, SWE 3- Shear wave elastography with first three measurements

Study	Severity of fatty liver	Index test	Threshold	SN	SP	PPV	NPV	AUC	p
Park et al. [13]	F	MRE	2.65	76.5	79.1	81.3	73.9	0.82 (0.74-0.91)	TE vs MRE =0.0116
	Stage 1-4 (n=51)	TE	6.1	66.7	65.1	69.4	62.2	0.67 (0.56-0.78)	
	versus								
	stage 0 (n=43)								
	NASH (n=72)									MRE	2.53	63.9	68.2	86.8	36.6	0.70 (0.57-0.82)	TE vs MRE =0.0011
		TE	5.6	61.1	59.1	83	31.7	0.35 (0.22-0.49)									
	Qu et al. [19]	F ≥ F2	LSM by TE	9.4	0.58 (0.47–0.68)	0.82 (0.72–0.92)	0.83 (0.73–0.93)	0.56 (0.45–0.66)	0.71 (0.63–0.80)	-
F ≥ F3		9.4		0.68 (0.55–0.81)	0.72 (0.63–0.81)	0.58 (0.45–0.70)	0.80 (0.72–0.89)	0.71 (0.62–0.80)	-
F = F4		11		0.80 (0.45–1.00)	0.71 (0.63–0.79)	0.09 (0.006–0.18)	0.99 (0.97–1.00)	0.77 (0.58–0.97)	-
Imajo et al. [23]	F≥1	MRE	2.92	78.2	100	100	82.4	0.947(0.863–0.980)	-
		VCTE	5	91.4	100	100	64.3	0.952(0.910–0.974)	-
		2D-SWE	6.35	82.5	100	100	80.5	0.923(0.851–0.962)	-
	F≥2	MRE	3.19	90.1	81.3	92.4	78.5	0.927(0.866–0.961)	-
		VCTE	8.4	86	74.2	89.4	67.6	0.882(0.823–0.931)	-
		2D-SWE	7.55	87.1	85.9	93.8	73.1	0.910(0.843–0.951)	-
	F≥3	MRE	3.9	82.5	91.5	91.7	82.1	0.937(0.882–0.958)	-
		VCTE	9.7	83.6	83.3	85.1	81.7	0.924(0.867–0.947)	-
		2D-SWE	8.88	87	87.8	89.3	84.9	0.920(0.865–0.953)	-
	F≥4	MRE	4.62	95.2	75	46.5	98.5	0.923(0.871–0.955)	-
		VCTE	12.4	90.2	74.6	45.1	97.1	0.872(0.807–0.917)	-
		2D-SWE	9.98	91.9	75.5	46.6	97.6	0.886(0.836–0.925)	-
Sharpton et al. [20]	Any fibrosis (F1–F4)	VCTE	7.8	64.4	87.8	90.4	58.1	0.81 (0.73–0.89)	-
		2D-SWE	7.5	53.4	90.2	90.7	52.1	0.72 (0.62–0.81)	0.03
	Significant fibrosis (F2–F4)	VCTE	6.8	94.6	62.3	54.7	96	0.86 (0.80–0.93)	-
		2D-SWE	7.7	75.7	85.7	71.8	88	0.84 (0.76–0.92)	0.5
	Advanced fibrosis (F3–F4)	VCTE	8.7	95	80.9	51.4	98.7	0.91 (0.82–0.99)	-
		2D-SWE	7.7	90	77	46.2	97.3	0.88 (0.81–0.96)	0.6
	Cirrhosis (F4)	VCTE	10.6	100	80	30	100	0.96 (0.91–1.0)	-
		2D-SWE	9.3	88.9	84.8	33.3	98.9	0.93 (0.86–0.99)	0.1
Taibbi et al. [15]	Significant fibrosis (F2–F4)	TE	≥7.9	63	63.2	-	-	0.719( 0.572-0.867)	0.016
		SWE-10	≥8.4	74	73.7	-	-	0.787(0.646-0.927)	0.002
		SWE-5	≥7.8	77.8	73.4	-	-	0.809(0.676-0.942)	0.001
		SWE-3	≥7.8	66.7	63.2	-	-	0.714(0.560-0.869)	0.021
	Advanced fibrosis (F3–F4)	TE	≥8.5	77.8	78.6	-	-	0.799(0.646-0.952)	<0.001
		SWE-10	≥9.1	72.2	78.5	-	-	0.797(0.659-0.935)	<0.001
		SWE-5	≥8.8	77.8	75	-	-	0.809( 0.684-0.933)	<0.001
		SWE-3	≥8.2	66.7	71.4	-	-	0.736(0.587-0.885)	<0.003
Tang et al. [21]	Center 1 analyst								
	0 (n = 58) vs. ≥ 1 (n = 33)	MRE	2.99 kPa	68	94	91	76	0.834(0.734, 0.912)	-
	≤ 2 (n = 73) vs. ≥ 3 (n = 18)	MRE	3.60 kPa	93	95	78	99	0.939(0.843, 0.997)	-
	Center 2 analyst								
	0 (n = 60) vs. ≥ 1 (n = 31)	MRE	2.98 kPa	66	96	94	75	0.833(0.745, 0.912)	-
	≤ 2 (n = 73) vs. ≥ 3 (n = 18)	MRE	3.65 kPa	93	95	78	99	0.947(0.856, 0.997)	-
Zhang et al. [22]**	Fibrosis stage 0 vs. 1–4, Stage 0 = 43; stage 1–4 = 57	MRE	2.01 kPa (2.60 kPa)*	0.912 (0.579)*	0.488 (0.907)*	0.703 (0.892)*	0.808 (0.619)*	0.81 (0.72–0.89)	-
		SWE	1.27 m/s (1.75 m/s)*	0.912 (0.333)*	0.116 (0.907)*	0.578 (0.826)*	0.5 (0.506)*	0.65 (0.54–0.76)	-
	Fibrosis stage 0–1 vs. 2–4, Stage 0–1 = 79; stage 2–4 = 21	MRE	2.77 kPa (3.06 kPa)*	0.905 (0.810)*	0.848 (0.911)*	0.613 (0.708)*	0.971 (0.947)*	0.94 (0.89–1.00)	-
		SWE	1.49 m/s (1.79 m/s)*	0.905 (0.476)*	0.43 (0.911)*	0.297 (0.588)*	0.944 (0.867)*	0.81 (0.71–0.91)	-
	Fibrosis stage 0–2 vs. 3–4, Stage 0–2 = 84; stage 3–4 = 16	MRE	2.77 kPa (3.17 kPa)*	0.938 (0.813)*	0.810 (0.905)*	0.484 (0.619)*	0.986 (0.962)*	0.95 (0.89–1.00)	-
		SWE	1.46 m/s (1.78 m/s)*	0.938 (0.625)*	0.393 (0.905)*	0.227 (0.556)*	0.971 (0.927)*	0.85 (0.74–0.96)	-
	Fibrosis stage 0–3 vs. 4, Stage 0–3 = 94; stage 4 = 6	MRE	2.77 kPa (3.42 kPa)*	1 (0.667)*	0.734 (0.904)*	0.194 (0.308)*	1 (0.977)*	0.92 (0.83–1.00)	-
		SWE	1.59 m/s (1.81 m/s)*	1 (0.833)*	0.617 (0.904)*	0.143 (0.357)*	1 (0.988)*	0.91 (0.79–1.00)	-
Ali AH et al. [24]	F ≥ 2	TE	12.8 kPa	73.70%	70.90%	24.60%	95.50%	0.723 (0.62–0.83)	-

Discussion

Due to the progressive nature of the NAFLD, it is of dire importance to diagnose early and prevent complications like cirrhosis, cancer, and cardiovascular disease. Of the different diagnostic modalities involved, imaging techniques are widely used, particularly ultrasonography and, in recent times, elastography. This systematic review aimed to compare the accuracy of ultrasonography and elastography by assessing the parameters of diagnostic test accuracy like sensitivity, specificity, AUROC, PPV, and NPV.

The conventional ultrasound helps estimate hepatic steatosis by assessing sure ultrasonographic signs like the brightness of the liver, visualization of intrahepatic vessels, and diaphragm [25]. A study conducted in a cohort of 72 patients of the geriatric age group found ultrasonography to have a sensitivity of 96%, specificity of 94%, and a positive predictive value of 98% to detect hepatic steatosis compared to MRS [14]. The combined score, in particular, showed higher accuracy when compared to the individual ultrasound criteria [14]. In a recent meta-analysis of twelve studies involving 2,921 participants, conventional ultrasonography's overall sensitivity and specificity for detecting ≥5% histologically defined HS were 82% and 80%, respectively [26]. Though the subjective assessment using ultrasonography has a low reproducibility [27], it can still be used for detecting moderate to higher steatosis grades due to its non-invasiveness, low radiation, affordability, and widespread use.

The advent of quantitative ultrasonographic methods like CAP and AC has helped to assess hepatic steatosis and fibrosis by measuring the ultrasound attenuation rate [28]. Beyer et al. found CAP to have higher accuracy in detecting lower levels of fat (steatosis ≥ 1) compared to higher levels (steatosis ≥ 2 and ≥ 3) [17]. It was also found that CAP could be confounded by body size, particularly in obese people. The study was limited by the participants' different geographical locations and eligibility criteria as the data was pooled from two independent studies [17]. A meta-analysis of nine studies, including 1,297 patients, showed that CAP had low accuracy for detecting severe grades of steatosis but had better performance for the S1 and S2 [29]. Though CAP has limitations, particularly in obese people and in higher grades of steatosis, and it needs more standardized cut-offs [ 25], it could be further improved upon and validated in further studies and represents a viable alternative non-invasive method for diagnosing hepatic steatosis.

The difference in which the steatotic and normal liver attenuates acoustic waves is how the AC measures are quantified [28]. Ogino et al. found a positive correlation r = 0.81, P < 0.01 between the AC values and LFC% (liver fat content%), and also good diagnostic accuracy scores for steatosis [18]. Nevertheless, with the progression of fibrosis, AC values were found to be decreasing. The results from this study were limited by its small sample size [18].

A study by Qu et al. evaluated the diagnostic performance of ultrasound attenuation parameter (UAP) and liver stiffness measurement (LSM) using Fibrotouch [19]. The diagnostic accuracy of FibroTouch (Kerry Medical Limited, Hong Kong, China) in the study was found to be higher in quantifying liver fat as the algorithm reduces the effect of subcutaneous fat on CAP computation. There was also a positive correlation between the LSM and degree of fibrosis and the UAP and steatosis [19]. For NAFLD, the area under the curve (AUC) values were found to be lower; hence, further validation studies may be needed [19].

Taibbi et al. showed that the diagnostic performances of shear wave elastography (SWE) 10, SWE 5, and SWE 3, compared with TE, had no significant difference in both significant and advanced fibrosis. However, it was higher for SWE 5 and SWE 10 [15]. Sharpton et al. found the diagnostic accuracy for detecting fibrosis lower for 2D SWE compared to VCTE, and this finding was particularly highlighted in those with higher BMI [20]. In a meta-analysis of nine studies with pSWE and 11 studies with TE, the diagnostic accuracy of both to detect advanced fibrosis and cirrhosis showed AUC of 0.94 and 0.95 for ≥F3 and =F4 (pSWE), and 0.92 and 0.94 for ≥F3 and =F4 (TE) [30]. When comparing SWE to MRE for staging fibrosis in patients with NAFLD, Zhang et al. found MRE to have more accuracy for earlier (≥ 1 and ≥ 2) fibrosis stages [22]. The AUC values of SWE were found to be lower for fibrosis staging here, and the study was limited by its small sample size and the cohort distribution of milder NAFLD [22]. Ali et al. found TE to be better at detecting fibrosis stage ≥2 when using an LSM cut-off value of 12.8 Kpa. He also found the diagnostic accuracy to increase when hemoglobin A1C (HbA1c) and alkaline phosphatase (ALP) were added to the LSM [24].

Park et al. showed MRE to have superior diagnostic accuracy compared to TE in diagnosing any stages of fibrosis except cirrhosis [13]. The negative predictive value was high for TE in diagnosing fibrosis (stages 2-4), (stages 3-4), and cirrhosis [13]. While the well-characterized prospective cohort served as its strength, this study was limited by its cross-sectional design and the median time interval of 107 days [13].

For detecting stage four fibrosis, Imajo et al. found MRE to be better than 2D SWE and VCTE, while the difference was less in stages ≥1, ≥2, and ≥3 [23]. The factors in the study which played a significant role in the discordance between 2D SWE, VCTE, and histopathology findings include skin capsule distance (SCD), sex, and interquartile range of liver stiffness to the median (IQR-median) [23]. There were no such factors affecting MRE. The interobserver and intraobserver repeatability was found to be excellent for MRI compared to 2D SWE and VCTE [23]. Tang A et al. found a high reproducibility when comparing the MRE liver stiffness from two centers with intraclass correlation coefficient (ICC) of ≥ 0.941, pairwise biases of ≤ 0.11 kPa, and reproducibility coefficients (RDCs) of ≤ 22.8% [21]. The study's limitations are the uneven distribution of liver fibrosis among the patient cohort and the inclusion of only one experienced analyst in the two academic centers [21]. In a meta-analysis of twelve studies including 910 patients, MRE was found to have diagnostic accuracy with AUROC of 0.89, 0.93, 0.93, and 0.95 for stages F ≥ 1, F ≥ 2, F ≥ 3, and F ≥ 4 [31]. MRE's high diagnostic accuracy and reproducibility in classifying liver fibrosis have provided an almost comparable method to liver biopsy. Nevertheless, the high cost and accessibility limit its widespread application. 

Limitations

This review included studies limited to the English language from mainly three databases from 2013-2021. Grey literature was not included. There was also heterogeneity in the included studies. The studies varied regarding the study population, index tests used, their cut-offs, and the reference standard. An analysis of the diagnostic accuracy could not be done due to the few studies included for the individual index test. Only one study evaluating qualitative ultrasound was included in the study. The degree of fibrosis in the study cohorts is also variable, which can affect the applicability of the results. Hence this review recommends prospective and cross-sectional studies with larger sample sizes and the same reference standard and index tests in the same population.

## Conclusions

In patients with NAFLD/NASH, MRI was found to be overall superior compared to other tests in terms of MRI PDFF for detecting steatosis or MRE for liver stiffness. However, its widespread application is limited by the high cost and accessibility. While quantitative ultrasonographic parameters have greatly improved the accuracy for detecting steatosis, and TE and pSWE are moderately effective in diagnosing fibrosis, there is insufficient data to arrive at a definite conclusion. Hence this review recommends the need for larger prospective or cross-sectional studies with the same reference standard and index test along with standardized cut-offs to improve the results' generalizability.
